# Discrimination of Different Breast Cell Lines on Glass Substrate by Means of Fourier Transform Infrared Spectroscopy

**DOI:** 10.3390/s21216992

**Published:** 2021-10-21

**Authors:** Maria Lasalvia, Vito Capozzi, Giuseppe Perna

**Affiliations:** Dipartimento di Medicina Clinica e Sperimentale, Università di Foggia, 71122 Foggia, Italy; maria.lasalvia@unifg.it (M.L.); vito.capozzi@unifg.it (V.C.)

**Keywords:** FTIR micro-spectroscopy, breast cell lines, principal component analysis

## Abstract

Fourier transform infrared (FTIR) micro-spectroscopy has been attracting the interest of many cytologists and histopathologists for several years. This is related to the possibility of FTIR translation in the clinical diagnostic field. In fact, FTIR spectra are able to detect changes in biochemical cellular components occurring when the cells pass to a pathological state. Recently, this interest has increased because it has been shown that FTIR spectra carried out just in the high wavenumber spectral range (2500–4000 cm^−1^), where information mainly relating to lipids and proteins can be obtained, are able to discriminate cell lines related to different tissues. This possibility allows to perform IR absorption measurements of cellular samples deposited onto microscopy glass slides (widely used in the medical environment) which are transparent to IR radiation only for wavenumber values larger than 2000 cm^−1^. For these reasons, we show that FTIR spectra in the 2800–3000 cm^−1^ spectral range can discriminate three different cell lines from breast tissue: a non-malignant cell line (MCF10A), a non-metastatic adenocarcinoma cell line (MCF7) and a metastatic adenocarcinoma cell line (MDA). All the cells were grown onto glass slides. The spectra were discriminated by means of a principal component analysis, according to the PC1 component, whose values have the opposite sign in the pairwise score plots. This result supports the wide studies that are being carried out to promote the translation of the FTIR technique in medical practice, as a complementary diagnostic tool.

## 1. Introduction

In recent years, Fourier transform infrared (FTIR) micro-spectroscopy has been held in increasing consideration as a versatile tool for biomedical and bioanalytical applications. The main reason for the interest in such a technique is that it provides biochemical information without employing reagents and has easy sample preparation. As a consequence, many works have proposed the FTIR technique as a complementary spectroscopic method to support diagnostic investigation of cytological samples [1,2,3,4,5]. In addition, FTIR has also been considered in clinical practice for biological tissues [6,7,8] and biofluids [9,10,11] analysis, as well as to monitor the response of cells and tissues to radiotherapy [12] and chemotherapy [13] treatments. 

In addition to FTIR, other examples of spectroscopy techniques able to provide biochemical information in a label free away are fluorescence [14] and Raman spectroscopy [15]. In particular, the Raman technique has been successfully proposed for cancer diagnostic applications of cytological [16], tissue [17] and biofluids [18,19] samples. FTIR and Raman techniques are considered complimentary methods to provide biochemical information about biological samples. The former technique is based on the selective absorption of incident IR radiation, whereas the latter one analyzes the inelastic scattering of incident light. The Raman spectroscopy has a few limitations, such as a longer time for measuring a spectrum with respect to FTIR, the possible damage to the sample (because the excitation beam consists of a laser focused on the sample) and the fluorescence from the sample which can strongly interfere with the Raman signal. Although FTIR spectroscopy has several disadvantages compared with the Raman technique, such as the worst spatial resolution and the impossibility of measuring samples in aqueous environment, it is preferable for diagnostic purposes, especially for the capability of providing reliable information in a relatively short time. 

One of the main obstacles to overcome before the adoption of the FTIR technique in cytological diagnostics is related to the substrate on which the cells must be deposited for the spectral measurements. In fact, the cell substrate most widely used by pathologists is a glass slide, with a thickness of about 1 mm. The advantage of these slides is that they are cheap, robust, and largely available. However, glass slides absorb IR radiation in the spectral range corresponding to the fingerprint region (1000–1800 cm^−1^), so making impossible the FTIR spectrum acquisition in this important spectra range, characterized by peaks related to the most important cellular components (proteins, lipids and nucleic acids). Nonetheless, glass substrates are transparent to mid-IR radiation in the high wavenumber spectral range (2000–4000 cm^−1^), where absorption peaks mainly related to lipid and protein components are located.

We recently showed that FTIR measurements in the high wavenumber spectral range are able to discriminate two cellular samples from different human cell lines, regardless of the growth substrate [20]. In particular, the FTIR spectra of the two cell lines were discriminated through the difference of the score values by performing a principal component analysis (PCA), which is a multivariate statistical technique widely used to discriminate spectral data [21]. Briefly, PCA transforms the N original variables, consisting in the absorption values for the N wavenumber values for the group of all the M measured samples, into a new set of N variables, called principal components (PCs), each one is a linear combination of the N original variables. Each original spectrum takes specific values in the set of PCs: such values are called scores. The criterion according to which the first PC is chosen is that it contains most of the variance of the scores, and each subsequent PC contains less variance. A score plot, reporting the score values of two different PCs for all the M samples, allows to visualize differences and similarities among the M samples, based on the original spectral characteristics.

Other authors successfully discriminated different types of cell lines, deposited on glass thick slides and thin coverslips, according to their FTIR spectra in the high wavenumber range and partially in the fingerprint range [22,23]. However, the cell lines investigated by these authors, as well as those measured by us in our previous paper [20], were very different among them. In particular, three types of cells were investigated by Rutter et al., i.e., cells from lung cancer (CALU-1 line), leukemia (K562 line), and peripheral blood (PBMC) [22,23]. Instead, we compared FTIR spectra of human neuroblastoma cells (SH-SY5Y line) with those of human normal keratynocytes cells (HUKE line).

In this paper, we investigate by FTIR micro-spectroscopy in the high wavenumber range three different samples of cells from breast tissue: non-malignant (MCF10A), malignant non-metastatic (MCF7), and metastatic (MDA). All the cellular samples were grown on conventional glass slides. We found that PCA technique is able to discriminate the spectra of the three samples according to the values of PC1 score. Although we found that the different relative amount of lipid component plays a fundamental role in the separation of score values of PC1, we remark that the principal component analysis was performed by considering the whole spectra of the samples (i.e., it was not limited to specific peaks). Therefore, full spectral data can be considered as spectral biomarkers of the cell type, without the need to search for any specific biochemical component. These results, obtained with cheap conventional glass slides, are promising for supporting the use of the FTIR technique as a complementary diagnostic tool in medical practice.

## 2. Materials and Methods

### 2.1. Cell Culture and Preparation

Three different breast cell lines, including non-malignant (MCF10A), malignant (MCF7), and metastatic (MDA-MB-231) cells were grown. MCF10A were cultured in DMEM/Ham’s F-12 (Sigma-Aldrich, Milano, Italy) supplemented with 100 ng/mL cholera toxin, 20 ng/mL EGF epidermal growth factor, 0.01 mg/mL insulin, 500 ng/mL hydrocortisone, and 5% horse serum (Life Technologies, Monza, Italy). MCF7 and MDA-MB-231 cells were cultured in the DMEM medium (DMEM, Life Technologies, Monza, Italy) and supplemented with 2 mM L-glutamine, (Sigma-Aldrich, St. Louis, MO, USA), 10% heat-inactivated fetal bovine serum (FBS) (Thermo Scientific), 1% penicillin/streptomycin (Life Technologies, Monza, Italy) and 0.25 ug/mL amphotericin B. 

Cells were grown on poly-lysine coated glass microscopy slides (Fisher Scientific, Rodano, Italy). The slides were located at the bottom of petri dishes incubated at 37 °C, and 5% CO_2_. Before FTIR measurements, the cells were fixed by means of 3.7% PFA in PBS solution and preserved inside a desiccator.

### 2.2. FTIR Measurements and Spectral Analysis

A FTIR Microscope HYPERION 2000 (Bruker Optik GmbH, Ettlingen, Germany) was employed to perform FTIR spectra in the transmission mode. The IR radiation entering the microscope came from a Vertex 70 Bruker interferometer (Bruker Optik GmbH). The IR signal was measured by means of a mercury cadmium telluride (MCT) detector, cooled at liquid N_2_ temperature. Each spectrum was estimated as an average of 64 scans in the 2500–4000 cm^−1^ range, with a resolution of 4 cm^−1^. The IR radiation was focused with a 15× objective on a sampling area of about 80 × 80 μm size, including 3–4 cells of each type. The background signal was measured from a spatial region of the slide close to the sampling area, but without any cell. About 30 cells were measured for each type of cells. The data acquisition was performed by means of the Opus 6.5 software (Bruker Optik).

For each FTIR measurement, the spectral range between 2750 and 3700 cm^−1^ was analyzed, because it includes the main spectral peaks related to radiation absorption from the cellular lipid and protein components. The spectra were normalized using standard normal variate (SNV) which, for each absorption intensity value corresponding to each wavenumber value, subtracts the mean value and then divides by the standard deviation value. Such a normalization procedure reduces spectrum baseline shifts due to scattering effects resulting from the interaction between IR radiation and sample particles [24] and minimizes the contribution of the absorption from cells having a different thickness. 

The R software was employed to achieve PCA by using the Chemospec package (version 3.4.1, R Core Team, Vienna, Austria, 2017) [25]. The t-test method was employed to evaluate the statistical differences between the groups of different cells, by means of the SigmaPlot software (version 12.5, Systat Software Inc., San Jose, CA, USA). 

## 3. Results and Discussion

**Figure 1:**
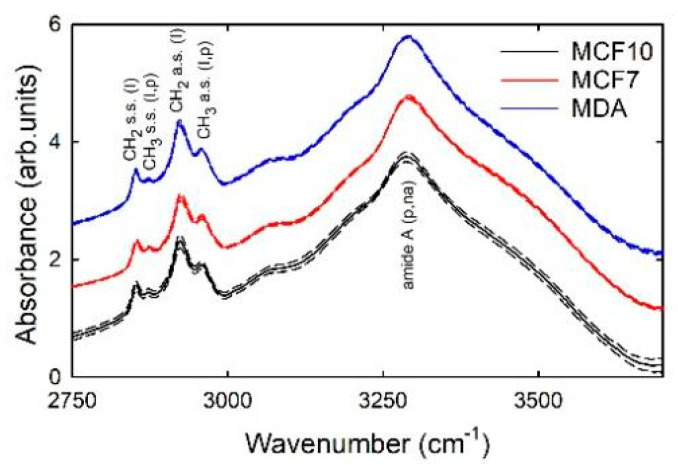
Mean FTIR spectra of MCF10A (continuous black line), MCF7 (continuous red line), and MDA (continuous blue line) cells after SNV normalization. Standard deviation spectra are also reported as dashed lines. The assignment of vibrational modes is also reported (c: carbohydrates; n.a.: nucleic acids; p: proteins; l: lipids). The spectra are vertically shifted for clarity of purpose.

The comparison among the normalized spectra of the three cell types grown on glass slides is shown in Figure 1, where the mean and standard deviation signals are reported. The mean spectra are not very different from each other, and they are also similar to the FTIR spectra obtained by other authors for breast cell lines and tissues [26,27,28]. Each spectrum is characterized by a few absorption peaks, which can be attributed according to the published literature [29]. Specifically, the broadest band at about 3300 cm^−1^ is related to the amide A (N-H stretching mode of proteins amino acids and nucleic acids), although a contribution of O-H stretching mode from residual water inside cells cannot be excluded. In addition, the peaks at about 2958 and 2871 cm^−1^ are due to the asymmetric and symmetric stretching mode of the CH_3_ groups of cellular proteins and lipids, respectively, whereas the peaks at 2924 and 2852 cm^−1^ can be attributed to the asymmetric and symmetric stretching of the CH_2_ groups of lipids, respectively.

Two main characteristics are evident in Figure 1. First, the standard deviation values suggest that the spectral intensities for the MCF10A cells spectra are more broadly distributed with respect to those of the other two cell lines, whose distributions of spectra are quite narrow. As the spectral variability is related to the biochemical content variability, we might deduce that non-malignant cells present larger differences of the relative content of cellular components with respect to the malignant and metastatic cancer cells. In addition, for each spectrum the FTIR signal between 2750 and 2850 cm^−1^ has a slightly larger intensity with respect to the signal at about 3700 cm^−1^, although no specific absorption peaks are reported for both such spectral ranges: this means that a baseline signal is still present. Therefore, the SNV normalization fails to totally remove the scattering signal.

**Figure 2:**
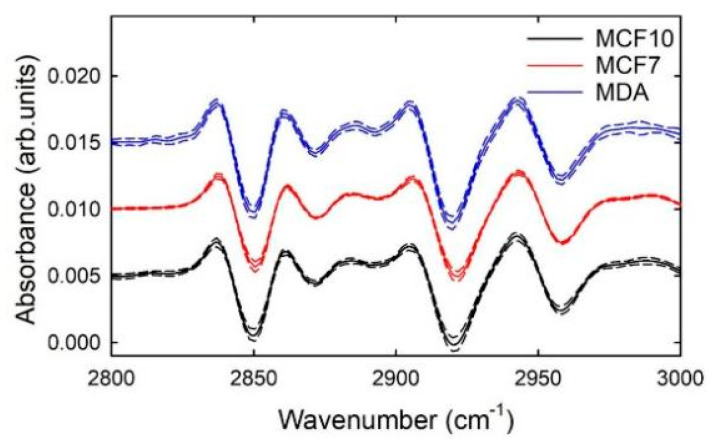
Mean second derivative signals of the SNV normalized FTIR spectra of MCF10A (continuous black line), MCF7 (continuous red line), and MDA (continuous blue line) cells. Standard deviation spectra are also reported as dashed lines. The spectra are vertically shifted for clarity of purpose.

In order to decrease the effect of the unwanted baseline and improve the comparison among the three cellular samples, second derivative signals of the SNV normalized spectra were calculated and are reported in Figure 2 as far as the 2800–3000 cm^−1^ spectral range is concerned. The subsequent analysis was focused on this spectral range, for two reasons: first, because the most interesting signals related to protein and lipid components are located in such a spectral range. Secondly, because of the doubts about the attribution of large a band at about 3300 cm^−1^, due to the uncertainty about the water contribution. Indeed, an important effect of the derivative process is that the signals of broad bands are suppressed relatively to those of sharp bands. So, since the scattering component resembles a very broad absorbance, using derivatives reduces the scattering effect. In particular, the second derivative was used because the most characteristic feature of a second-order derivative is a negative band with a minimum at the spectral position corresponding to the maximum on the zero-order band. An interesting feature visible in Figure 2 is that the intensity signals corresponding to the minima of the derivative spectra of the three cell lines show similar variability (as deduced by standard deviation values). By considering what was reported above for the comparison of the SNV spectra in Figure 1, it can be deduced that the largest variability of MCF10A cells is probably related to the distribution of the size of the scattering centers, which are the nuclei for cellular samples [30].

**Figure 3:**
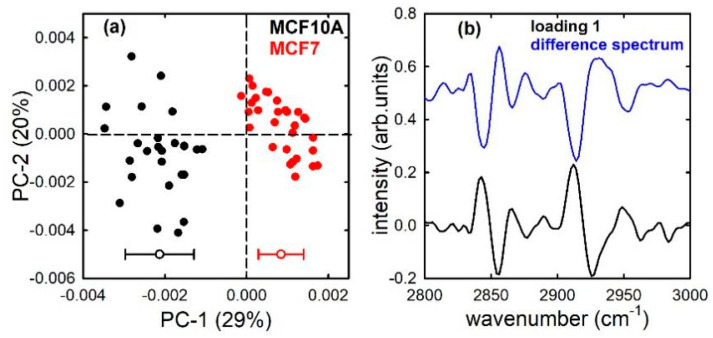
PC1 and PC2 score plot (**a**) for the MCF10A (black dots) and MCF7 (red dots) cells; average (−0.0021 and +0.0008 for MCF10A and MCF7, respectively) and standard deviation (0.0008 and 0.0005 for MCF10A and MCF7, respectively) of the PC 1 distribution values are shown at the bottom of (**a**). Loading 1 spectrum (black line), compared with the difference between average spectra (blue line), is reported in (**b**).

The PCA technique was used to assess whether FTIR is able to discriminate between the non-malignant MCF10A cells and the two types of mentioned cancer cells. Figure 3a shows the PCA score plots for the MCF10A and MCF7 cells: it is clearly visible that the two types of cells are separated according to the PC1 component. In fact, MCF10A scores (black dots) are characterized by negative values, whereas MCF7 scores (red dots) have mainly positive values. The average and standard deviation values of scores distributions are reported at the bottom of Figure 3a. The differences of PCA score values between the two types of cells were statistically significant, as confirmed by a *t*-test yielding a *p*-value < 0.001. The loading 1 plot, shown in Figure 3b (black line), is in good agreement with the difference plot (blue line) between the average spectra of the two types of cells (considering that spectral positions of positive and negative peaks are exchanged). In particular, the largest negative bands in the loading 1 plot are located at about 2855 and 2925 cm^−1^, corresponding to the spectral positions of CH_2_ stretching modes of lipid components in Figure 1. The loadings represent coefficients describing the influence of the variables (IR absorption at specific wavenumber values) on the score values for a given PC [31]; therefore, Figure 3b suggests that the spectral origin of the variations which differentiate the two types of cells is related to the different relative amount of lipid components. Such a result is in agreement with the results reported by C. Nieva et al. [32], by using the Raman micro-spectroscopy and immunocytochemistry methods. They found that the lipid content in MCF10A cells is larger than in MCF7 cells. Apart from this specific biochemical marker that differentiates the two types of cells, the results shown in Figure 3a suggests that the FTIR technique, associated with multivariate methods such as PCA, is able to discriminate the two cell types on the basis of a spectral marker, i.e., a combination of several peaks and bands that make up the whole spectrum in the measured wavenumber range.

**Figure 4:**
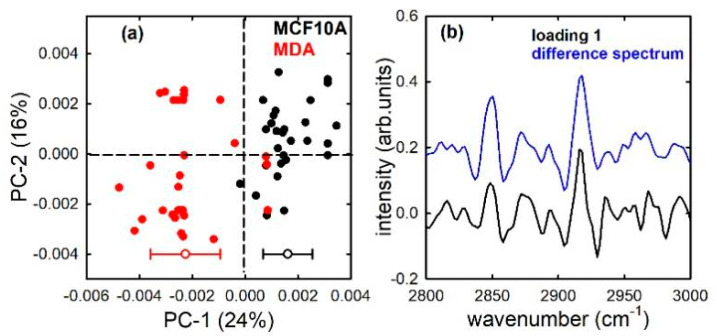
PC1 and PC2 score plot (**a**) for the MCF10A (black dots) and MDA (red dots) cells; average (+0.0016 and −0.0023 for MCF10A and MDA, respectively) and standard deviation (0.0009 and 0.0013 for MCF10A and MDA, respectively) of the PC 1 distribution values are shown at the bottom of (**a**). Loading 1 spectrum (black line), compared with the difference between average spectra (blue line), is reported in (**b**).

PC1 also discriminates MCF10A and MDA cells, as shown in the score plot Figure 4a, with the MCF10A cells corresponding mostly to positive score values and MDA cells mainly to negative values, although this separation is not as clear as in Figure 3a and some outliers are visible. Therefore, the FTIR spectrum in the high wavenumber range successfully can be considered as a spectral marker capable of separating the two types of cell without the need to investigate for any biochemical cellular component whose relative content is different for the two cell lines. However, the similarity of the loading plot to the difference of average spectra in Figure 4b suggests that the vibrational modes of lipids mainly contribute to the separation of the two cell lines. Small differences in the relative lipid content of MCF10A and MDA cells have been attributed to a large content of lipid droplets in MDA metastatic cells [32]. In fact, it is well-known that lipid metabolism is altered in metastatic cells, which use lipids as a source of energy to proliferate more and more [33,34].

**Figure 5:**
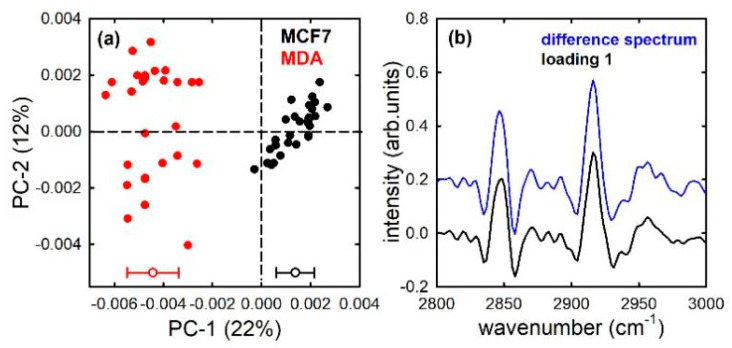
PC1 and PC2 score plot (**a**) for the MCF7 (black dots) and MDA (red dots) cells; average (+0.0014 and −0.0044 for MCF7 and MDA, respectively) and standard deviation (0.0008 and 0.0011 for MCF7 and MDA, respectively) of the PC 1 distribution values are shown at the bottom of (**a**). Loading 1 spectrum (black line), compared with the difference between average spectra (blue line), is reported in (**b**).

The PCA analysis performed for MCF7 and MDA cells is characterized by a large separation of the score values according to PC1, as shown in Figure 5a. In this case, from a biochemical point of view, the discrimination occurs as a consequence of the difference in the relative amount of lipid components, as suggested by the loading 1 and difference plots in Figure 5b. Previously reported findings also observed that MCF7 cells are characterized by a lower amount of fatty acids, both membrane phospholipids and cholesterol [32]. The lipidomic analysis performed by means of liquid chromatography mass spectrometry for these two different breast cancer cell lines showed that they are characterized by different mutual proportions of specific lipids [35]. However, we remark that Figure 5a shows that the FTIR spectra constitute a reliable marker for the spectral characterization of the cellular samples and for the differentiation of their different types.

**Figure 6:**
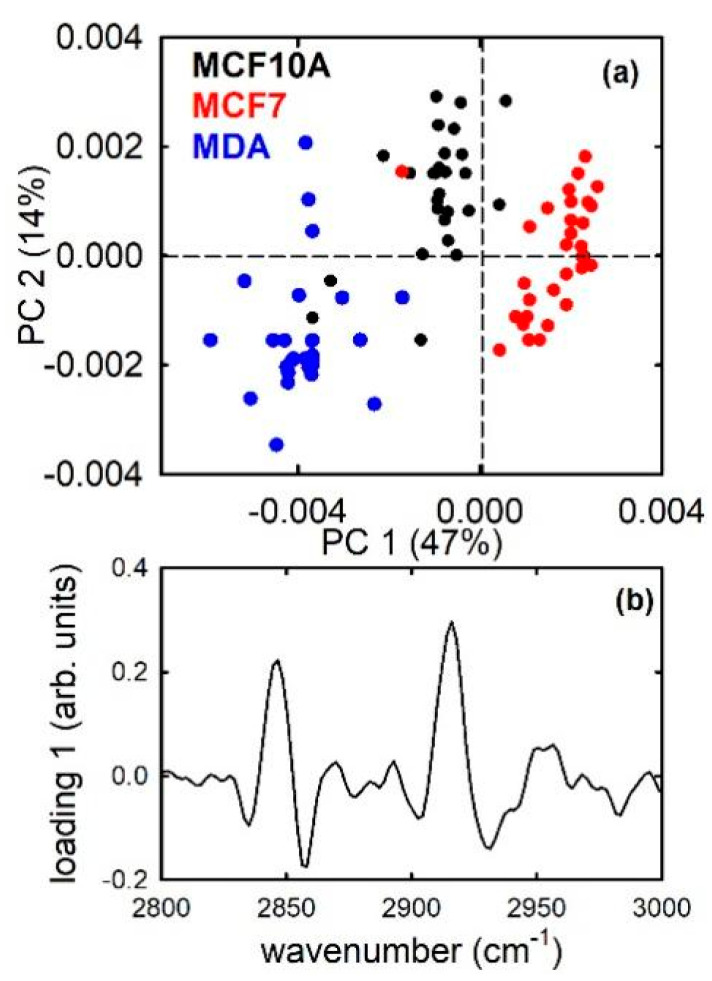
PC1 and PC2 score plot (**a**) for the MCF10A (black dots), MCF7 (red dots), and MDA (blue dots) cells and loading 1 spectrum (**b**).

If we consider the difference of average PC1 score values as an estimate of the discrimination quality, Figure 3, Figure 4 and Figure 5 suggest that non-malignant (MCF10A) cells can be better separated from metastatic (MDA) cells than from malignant non-metastatic (MCF7) cells. In particular, the difference of average PC1 score values is 0.0039 ± 0.0003 in the former case, and 0.0030 ± 0.0002 in the latter one. Moreover, the best separation occurs between MCF7 and MDA cells, whose difference of PC1 score values is 0.0058 ± 0.0002. This is probably related to the main role of lipid components in the discrimination among such cell lines: indeed, as described above, MCF7 cells are characterized by the smallest relative amount of lipid components [32]. Such different separation is also clearly visible in Figure 6, which shows the results of the PCA analysis of the three cell lines. Figure 6a confirms that PC1 score values are able to significantly discriminate (*p* < 0.001) the three types of cell line. In particular, PC1 scores are mainly positive for MCF7 cells and mostly negative for MDA and MCF10A cells, with the former cells having larger negative values than the latter ones. Figure 6b suggests that the separation between positive and negative values occurs because of the different lipid contents (the negative bands in Figure 6b are located at spectral positions corresponding quite to lipid peaks in the absorption spectra), as a consequence of the low lipid relative amount of MCF7 cells with respect to the other two types of cells.

Overall, the PCA analysis of FTIR spectra measured in the high wavenumber spectral range proved to be a reliable tool for discriminating non-malignant cells from malignant cells and from metastatic cells. The aspect to be remarked is that such separation can yield possible application in clinical diagnostics, even neglecting any discussion about the biochemical components.

## 4. Conclusions

The obtained result observe that FTIR spectra in the high wavenumber range and the principal components analysis technique are able to discriminate three cellular samples from different cell lines related to the same tissue. In particular, three breast cell lines were successfully separated: MCF10A, consisting of a non-malignant mammary epithelial cell line; MCF7, which is a human breast non-metastatic adenocarcinoma cell line; and MDA, which is a metastatic mammary adenocarcinoma cell line. The three cell samples, grown on conventional microscopy glass slides and measured by means of the FTIR technique in transmission mode, were discriminated according to PC1 score values. The separation occurs as a consequence of the different relative content of lipid components, as deduced by the features at 2924 and 2852 cm^−1^ in the loading plots, which are related to the asymmetric and symmetric stretching modes of the CH_2_ groups of lipids. 

Regardless of the biochemical point of view, a significant feature to remark is that FTIR spectrum in the 2800–3000 cm^−1^ range can be used as a spectroscopic biomarker for the discrimination of non-malignant, malignant, and metastatic breast cells. This means that the whole spectrum can be used as a marker of cell status, instead of specific spectral peaks (molecular markers). In addition, the use of the microscopy glass slide as cell growth substrates is worthy of attention because such slides are widely used in a clinical environment for cytological and histological diagnosis. Therefore, the obtained results suggest a possible implementation of the FTIR technique as a complementary diagnostic tool in medical practice.

However, it must be remarked that the PCA method, while displaying the discrimination between objects belonging to different classes, is not a classification method. Therefore, it does not allow to properly classify new objects (spectra, in our case) whose classification is unknown. For this purpose, appropriate statistical techniques must be used (e.g., k-nearest neighbors, soft independent modeling of class analogies, and support-vector machine). Hence, the PCA analysis is only a starting point for using FTIR in medical diagnostics.

In addition, there are also other limits and hindrances to be overcome before the use of FTIR in diagnostics can be accepted. First of all, an in-vivo analysis is difficult to perform. Being an in-vitro method, such a procedure is albeit minimally invasive, because it needs cellular samples to be extracted from patients. Further, it is necessary to also extract healthy cells from the patient if the aim is to discriminate healthy cells from pathological cells.

Other authors have shown the possibility to discriminate different types of cells grown onto a glass substrate by means of FTIR spectra in the high wavenumbers range [22,23]; however, they investigated cells from different tissues. Our findings point out that the discrimination is possible also for cells from the same type of tissue but at a different pathology stage. This is an interesting, but not decisive, step towards the introduction of this spectral investigation in the medical diagnostic field. Further steps are both to confirm the separation by using cell lines from other types of tissue than breast and particularly, to measure ex-vivo cells as well as tissues from patients.
